# Safety and feasibility of oral immunotherapy to multiple allergens for food allergy

**DOI:** 10.1186/1710-1492-10-1

**Published:** 2014-01-15

**Authors:** Philippe Bégin, Lisa C Winterroth, Tina Dominguez, Shruti P Wilson, Liane Bacal, Anjuli Mehrotra, Bethany Kausch, Anthony Trela, Elisabeth Hoyte, Gerri O’Riordan, Scott Seki, Alanna Blakemore, Margie Woch, Robert G Hamilton, Kari C Nadeau

**Affiliations:** 1 Allergy, Immunology, and Rheumatology Division, Stanford University, CCSR 3215, Stanford, CA 94305, USA; 2 Johns Hopkins University School of Medicine, Dermatology, Allergy and Clinical Immunology Reference Laboratory, Baltimore, MD, USA

**Keywords:** Food allergy, Oral immunotherapy (OIT), Specific oral tolerance induction (SOTI), Multiple, Safety, Efficacy

## Abstract

**Background:**

Thirty percent of children with food allergy are allergic to more than one food. Previous studies on oral immunotherapy (OIT) for food allergy have focused on the administration of a single allergen at the time. This study aimed at evaluating the safety of a modified OIT protocol using multiple foods at one time.

**Methods:**

Participants underwent double-blind placebo-controlled food challenges (DBPCFC) up to a cumulative dose of 182 mg of food protein to peanut followed by other nuts, sesame, dairy or egg. Those meeting inclusion criteria for peanut only were started on single-allergen OIT while those with additional allergies had up to 5 foods included in their OIT mix. Reactions during dose escalations and home dosing were recorded in a symptom diary.

**Results:**

Forty participants met inclusion criteria on peanut DBPCFC. Of these, 15 were mono-allergic to peanut and 25 had additional food allergies. Rates of reaction per dose did not differ significantly between the two groups (median of 3.3% and 3.7% in multi and single OIT group, respectively; p = .31). In both groups, most reactions were mild but two severe reactions requiring epinephrine occurred in each group. Dose escalations progressed similarly in both groups although, per protocol design, those on multiple food took longer to reach equivalent doses per food (median +4 mo.; p < .0001).

**Conclusions:**

Preliminary data show oral immunotherapy using multiple food allergens simultaneously to be feasible and relatively safe when performed in a hospital setting with trained personnel. Additional, larger, randomized studies are required to continue to test safety and efficacy of multi-OIT.

**Trial registration:**

Clinicaltrial.gov NCT01490177

## Introduction

Food allergy is the leading cause of fatal and recurring anaphylaxis in children and teenagers in both Europe and the United States [1-3]. The current standard of care is to practice strict avoidance of the food allergens and have injectable epinephrine readily available, in case of accidental exposure [1]. Unfortunately, unintentional ingestion is a common occurrence [4].

Oral and sublingual allergen-specific immunotherapies have been proposed as possible methods of desensitization and, possibly, of induction of tolerance, with several prior studies having shown some success in using these approaches for single specific food allergens such as milk [5-11], egg [10-14], peanut [15-20], and hazelnut [21]. These monotherapies appeared relatively safe when conducted in a supervised and controlled setting, with severe reactions requiring epinephrine being rare.

Despite these promising results, there is lack of information regarding simultaneous administration of multiple foods within the same treatment. This is an important caveat considering that 30% of food allergic participants under 18 years old are estimated to be allergic to more than one food [22-24]. This estimate has been reported to increase to 70% when considering highly atopic children [25]. Compared to those with single food allergies, these participants experience a greater decrease in quality of life [26], are more likely to suffer from dietary deficiencies [27] and are less prone to spontaneously outgrowing their allergies [28].

Since OIT relies on allergen ingestion on a daily basis, mostly at home, the main concerns with simultaneous allergen administration are about safety. Previous studies using non-specific anti-IgE stimulation showed that binding of only 200 to 500 of the 250, 000 surface IgE molecules on a basophil are required to trigger degranulation [29]. A concern is that administration of multiple allergens simultaneously would in theory result in an increased number of specific IgE molecules being simultaneously bound and cross-linked on mast cells and basophils, increasing the risk of reaching this threshold. It is also unknown whether such an approach would affect treatment efficacy. One could hypothesize that immunologic responses and memory responses to each food would be allergen specific; however, synergic effects cannot be excluded.

The primary endpoint (safety) of our investigation was the occurrence of allergic reactions throughout the course of the study, comparing food allergic participants with either peanut alone or multiple foods in their treatment.

## Methods

This phase 1 study was performed in a single center in a hospital setting with Institutional Review Board approval, under Investigational New Drug (IND) approval. Participant selection, study medication and design are described in the Additional file 1. Briefly, participants older than 4 years were eligible for inclusion if they had proven sensitivity to the food allergen documented by both a skin prick test greater than 7 mm (wheal) and specific IgE greater than 2kU/L to peanut as well as positive allergic reaction in a double-blind placebo-controlled oral food challenge (DBPCFC) up to a cumulative dose of 182 mg as per Bock’s criteria [30]. Further DBPCFC were also performed following the same protocol to nuts, sesame seed, dairy or egg to document additional food allergies. Exclusion criteria (which included severe anaphylaxis requiring ICU admission and poorly controlled asthma) are listed in the Additional file 1.

**Figure 1:**
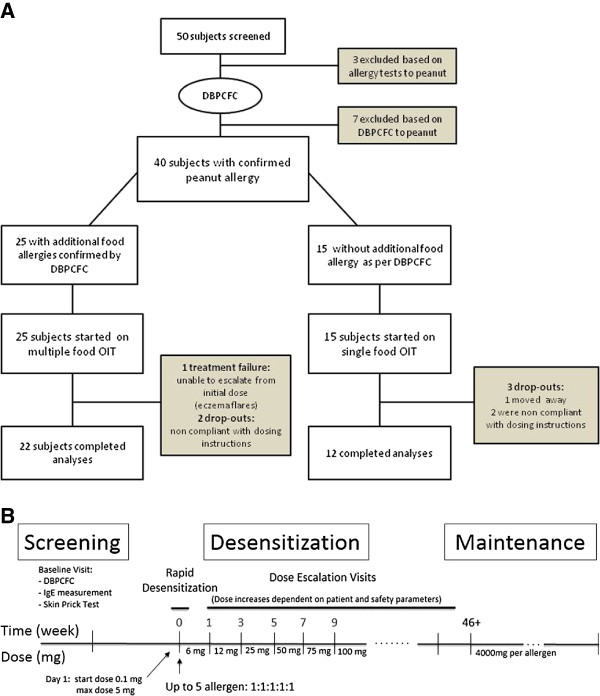
**OIT trial design including (A) screening and trial flow chart and (B) immunotherapy protocol timeline.** Amount of maintenance dose depends on number of allergen in mix (4000 mg per allergen).

Participants who reacted only to peanut on their inclusion DBPCFC were assigned to the single OIT group while those who reacted to additional foods were assigned to multiple food therapy (Figure 1A). The multi OIT regimen (up to five food allergens could be used) was customized to what the participant was found to be allergic to by DBPCFC. The OIT protocol for both groups (single OIT and multi OIT) consisted of three phases: (1) the initial escalation day (or modified rush day), (2) home dosing with biweekly visits for dose escalations and (3) the maintenance phase (Figure 1B) which are detailed in the Additional file 1. Participants were instructed to take oral cetirizine (dosed as per each product insert) 1 hour before home doses and trained on the use of epinephrine (see details in the Additional file 1). The primary goal of the OIT was to achieve a 10-fold increase from initial DBPCFC threshold. The dosing protocol was designed to continue dose increases up to a daily maintenance dose of 4000 mg protein of each allergen (up to 20,000 mg cumulative dose for those on 5 allergens).

### Serological analysis

Sera at baseline and at 12 months were analyzed for food-specific IgE and IgG4 levels by immunoCAP FEIA (Thermofisher Scientific/Phadia, Kalmazoo, MI) when available. IgE antibody levels < 0.1 kU_A_/L and IgG4 antibody levels <0.01 kU_A_/L were considered undetectable. IgE antibody levels between 0.1 and 0.35 kU_A_/L currently have an undetermined clinical significance.

### Statistical analysis

Clinical characteristics and safety data were compared between the groups using student T test for continuous and Pearson’s chi-square for dichotomic variables. Dose progression was measured as time to reach 10-fold increase from initial cumulative dose eliciting a reaction on DBPCFC to peanut as well as doses of 300, 1000 and 4000 mg protein per food allergen. Groups were compared with Kaplan-Meier curves using the Breslow test. Changes in serologies were assessed by Wilcoxon matched-pairs signed rank test.

## Results

A total of 40 participants ranging in age from 4 to 46 years met inclusion criteria on DBPCFC and were enrolled in an OIT protocol. Fifteen (15) with single peanut allergy documented by DBPCFC were assigned to peanut single OIT. The 25 remaining eligible participants received multi OIT for multiple allergens. Detailed allergen combinations in the multi group are available in the Additional file 1. Clinical characteristics of each group are compared in Table 1. There were no statistically significant differences between the groups. Both groups were also comparable with regards to their peanut allergy SPT, specific IgE and DBPCFC results. Allergy evaluation results for other foods included in the multi group are available in the Additional file 1.

**Table 1:** Baseline characteristics

	**Multiple food allergy group**	**Single peanut allergy group**
**Number of participants**	25	15
**Median Age in yrs. (range)**	8 (4–25)	10 (5–46)
**Male**	14 (56%)	8 (53%)
**Coexisting atopic disease**		
Atopic Dermatitis	17 (68%)	8 (53%)
Allergic Rhinitis	13 (52%)	9 (60%)
Asthma	17 (68%)	10 (66%)
**Baseline testing to peanut (median and range)**		
SPT in mm	15 (7–25.5)	12 (7–22)
Specific IgE in kU_A_/L	90.4 (2.43-100)	80 (3.66-100)
DBPCFC step eliciting symptoms (mg protein)	50 (0.1-100)	25 (1.6-100)
**Symptoms upon peanut DBPCFC**		
Skin	20 (80%)	12 (80%)
Upper airways	18 (72%)	12 (80%)
GI	17 (68%)	12 (80%)
Lower airways	6 (24%)	3 (20%)
**Other food allergies meeting DBPCFC criteria for inclusion**		
Walnut	14 (56%)	N/A
Cashew	13 (52%)	N/A
Pecan	7 (28%)	N/A
Milk	7 (28%)	N/A
Egg	6 (24%)	N/A
Sesame	6 (24%)	N/A
Almond	5 (20%)	N/A
Hazelnut	3 (12%)	N/A
**Number of food in mix**		
2	6 (24%)	N/A
3	8 (32%)	N/A
4	5 (20%)	N/A
5	6 (24%)	N/A

There were no serious adverse events in the study. Over the study period, there were 5 drop outs for reasons which included non-compliance with study medication (n = 4) and change of residence (n = 1). One participant in the multi OIT group was unable to increase doses due to eczema flares that prevented initial escalation and was categorized a treatment failure (n = 1). These participants were included in safety analyses but censored at the point where they were excluded. Overall, a total of 277 hospital escalation doses and 7,830 home doses were given in the peanut monotherapy group; while 603 hospital escalation doses and 12,030 doses were given to the participants receiving multi-allergen OIT (Table 2). No more than 3 doses were missed consecutively by any one participant, based on dose diary review.

**Table 2:** Reaction rates

	**Multi (n = 25)**	**Single (n = 15)**	**p-value**
**Initial escalation day**			
* Reactions (Reaction rate)*	15 (60%)	6 (40%)	**.22**
* Epinephrine use*	0	0	**1.00**
**Dose escalations**			
* Total doses*	603 doses	277 doses	
* Median reaction rate [range]*	3.4% [0–23.1]	3.7% [0–16.6]	**.31**
* Epinephrine use*	0	0	**1.00**
**Home dosing**			
* Total doses*	12030 doses	7830 doses	
* Median reaction rate [range]*	3.1% [0.6-29.2]	2.9% [0.1-59.0]	**.65**
* Epinephrine use (per dose)*	2 (0.02%)	2 (0.03%)	**.67**
* Epinephrine use (per participant)*	2 (8%)	2 (13%)	**.62**

**Figure 2:**
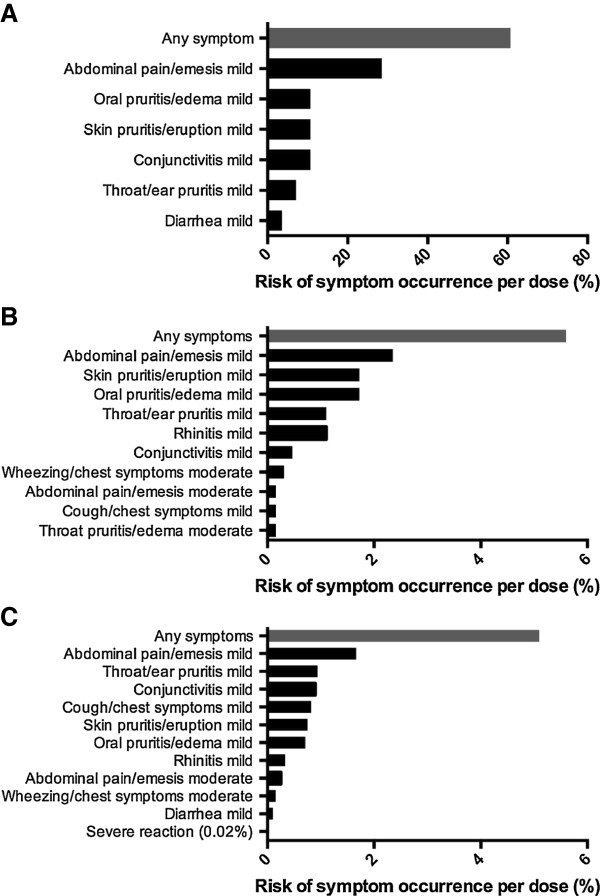
Symptom occurrence with (A) initial escalation day, (B) dose escalations and (C) home dosing during OIT to multiple foods.

Most reactions to multiple food OIT doses were mild, with abdominal pain being the most frequently reported symptom, both in hospital setting and at home. Figure 2 presents symptom profiles per doses while Table 2 reports distribution of participants’ individual reaction rates for each OIT phase. Most participants experienced symptoms on the initial escalation day (60%) which were mild. Our data showed no statistical difference in adverse event rate or severity when comparing customized multi OIT regimens (i.e. there were not higher rates of allergic reactions with particular or with greater number of foods in mix).

Participants in the monotherapy group had a similar reaction profile (Additional file 1: Figure S1). Of note, one patient in this group reported frequent mild abdominal cramping with 369 of 630 home doses (59%) (not shown in Additional file 1: Figure S1). She was able to progress normally with dose escalations and these reactions eventually subsided while on maintenance. Table 2 compares reaction rates in both groups, which did not differ significantly.

Two severe reactions requiring epinephrine occurred in each group after home dosing (Additional file 1: Table S3). In the monotherapy group, one participant had abdominal pain and wheezing within 20 minutes of food allergen ingestion and was immediately treated with injectable epinephrine; symptoms resolved within 6 minutes of treatment. Another participant had urticaria and wheezing within 40 minutes of food allergen ingestion and was immediately treated with injectable epinephrine; symptoms resolved within 5 minutes of treatment. In the multi-allergen group, one participant had wheezing and angioedema around the eyes within 25 minutes of food allergen ingestion, and the reaction resolved within 3 minutes following administration of injectable epinephrine. The other participant on multi OIT with a severe reaction had abdominal pain, urticaria, and wheezing within 35 minutes of food allergen ingestion and was immediately treated with injectable epinephrine; symptoms resolved within 6 minutes of treatment.

**Figure 3:**
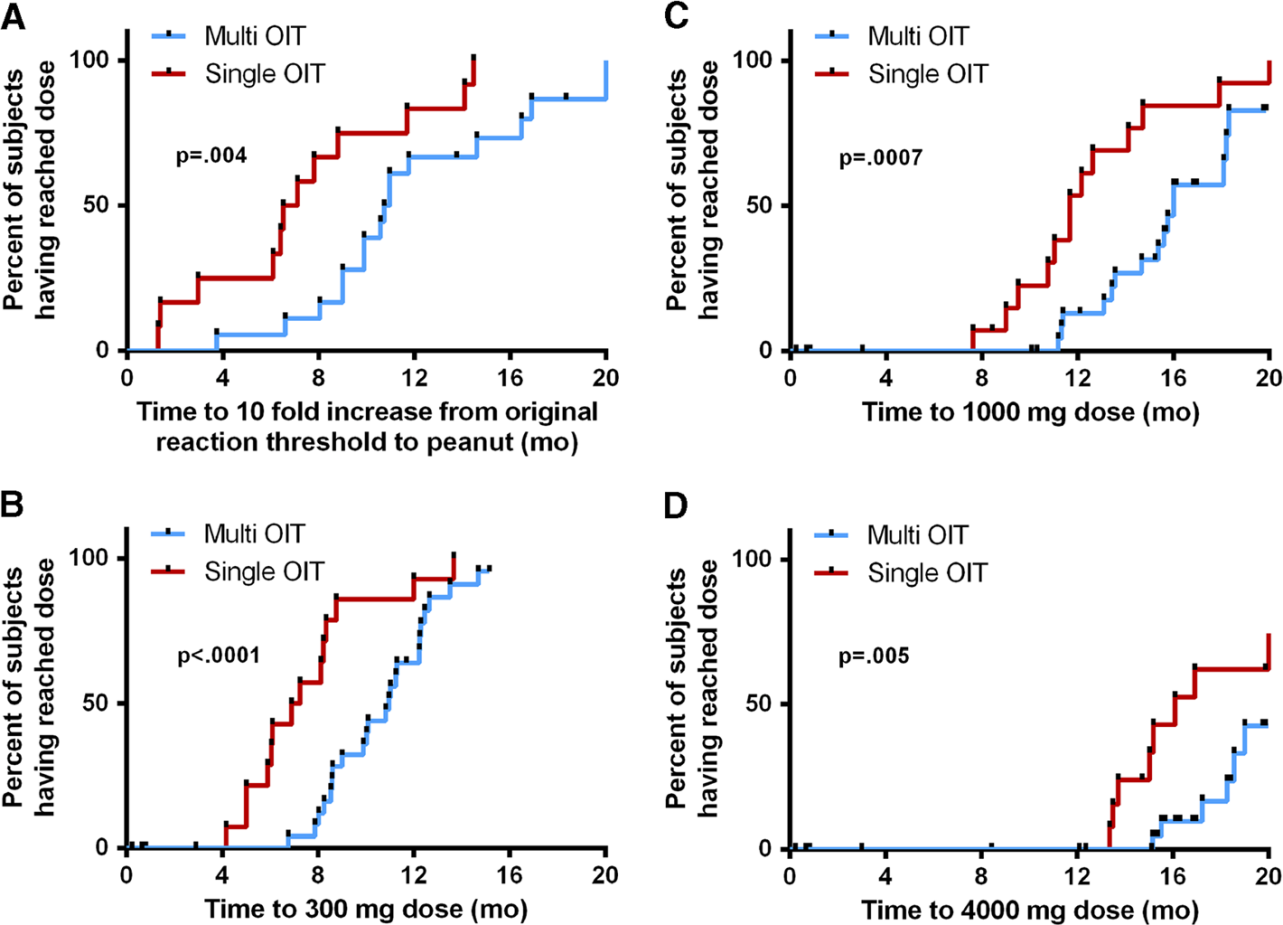
**Kaplan-Meier curves showing time to dose of 300 mg (A), 1000 mg (B), and 4000 mg (C) per allergen in mix.** Panel **D** shows time to reach the dose corresponding to a 10 fold increase from the threshold at which the patient reacted to peanut on initial DBPCFC. P-values from *χ*^2^ analysis were calculated using Breslow method.

Kaplan-Meier curves showing time to reach a 10-fold increase in threshold dose of food allergen protein, as well as time to reach a dose of 300 mg, 1000 mg and 4000 mg food allergen protein are presented in Figure 3. Participants undergoing monotherapy reached these 4 secondary endpoints significantly faster than those on multi-allergen OIT (p = .004, p < .0001, p = .0007, and p = .005, for respective outcomes) which was expected given each allergen represented only a fraction of the whole dose in the latter group. COX regression analysis showed no statistical difference in dose progression comparing number or combinations of foods in the OIT mix (data not shown).

**Figure 4:**
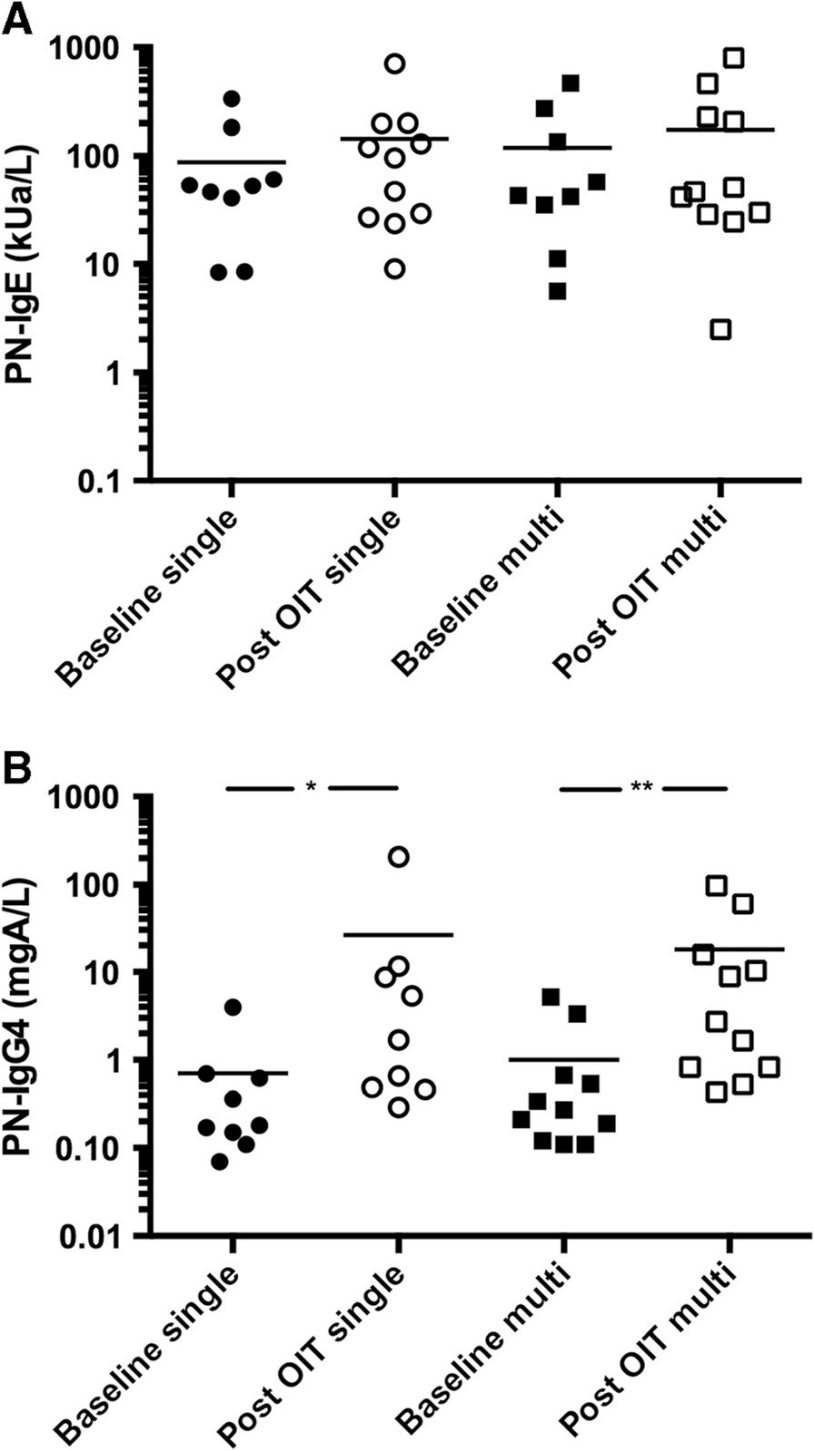
**Comparison of peanut-specific IgE (A) and IgG4 (B) at baseline and after one year of OIT.** *p = 0.001; **p = 0.008.

One year into OIT, peanut-specific IgE remained unchanged in both groups while peanut specific IgG4 antibody increased significantly (p = 0.001 and p = 0.008 in multi and single OIT respectively) (Figure 4). Similar trends were found to other foods in the multi OIT group (Additional file 1: Figure S2).

## Discussion

In this phase 1 study, we have shown that participants allergic to multiple foods can be safely desensitized to up to five foods simultaneously using a modified OIT protocol. Despite the increasing interest in food OIT in recent years, the safety or efficacy of using multiple food flour/powder allergens in parallel has, to our knowledge, not been published. These findings are particularly relevant considering the already high and likely growing number of food allergic participants who are allergic to more than one food allergen [22-25].

The multi OIT study was designed as a proof of concept, phase 1 study; therefore, safety measurements were the primary endpoint. The rate of reactions observed in the multi-allergen OIT group was within the acceptable range for an OIT study and was similar to a reference cohort of peanut mono-allergic participants undergoing the same protocol to peanut only. This supports the view that it is not the diversity or multiplicity of the food allergen binding surface IgE but rather the total dose of allergen administered that determines OIT reactions. However, this data should be viewed as proof of concept data until randomized, controlled, double-blinded phase 2 studies with larger sample sizes are performed.

In previous single OIT studies, overall reaction rates tended to vary, possibly due to differences in escalation protocols, allergens, selection of participants or use of anti-histamine pre-medication [7-9,13-15,19]. However, severe reactions needing epinephrine injections have been consistently shown to be an occurrence, albeit rare, when performing OIT. In our study, 2 participants from each group required epinephrine during the study period. Although the number of allergens did not seem to increase the risk of severe reactions, a state of continual vigilance is needed to perform OIT. As most reactions occurred at home (including those severe reactions requiring injectable epinephrine), participants and families carry reaction medications at all times and are educated on the proper use of injectable epinephrine and on the recognition of severe reactions that warrant its use. It is also important that the food doses be from a verifiable and reproducible source, that they be carefully measured and cross-checked by clinical staff and stored and dispensed from an appropriate facility.

We chose to proceed with a single dose of each food in an equal protein stoichiometric ratio. In some smaller children that are slow eaters, the process of eating their dose could take up to 1 hour with a mix of 5 nuts at full dose. In such cases, it would not have been feasible to wait in between foods for the occurrence of a reaction. Our final maintenance dose was 4000 mg per allergen. The optimal long term maintenance dose for food OIT has not been identified yet and may need to be individualized. More studies are needed to determine this parameter of OIT which may have an impact on subject compliance with ingestion.

Except for one participant who was excluded due to eczema flares and two drop-outs, all participants reached a 10 fold increase in their reaction threshold during the study period. The median time at which participants on single allergen OIT reached this dose was 14 weeks earlier than for those on the multi-allergen therapy. Participants undergoing multi-allergen OIT also took more time reaching the 300 mg, 1000 mg and 4000 mg doses. This delay is to be expected since there were up to 5 food allergens given simultaneously and the dose for each individual food allergen was divided evenly. Importantly, this phase 1 study demonstrates that it is possible and feasible to test the effect of multi food allergen therapy simultaneously, rather than performing single immunotherapy in sequence for patients, a process that could take many years for patients who are multi-sensitized to food allergens.

Although no SCORAD was calculated, it is worth noting that the participant that failed the initial dose escalation had significantly more severe eczema than other participants, for which he reported a history of systemic treatment and a clear relation with ingestion of food allergens. The eczema was active at enrolment despite topical treatment.

Despite showing proper dose progression, our study did not prove treatment efficacy. To measure true clinical tolerance, participants would have to stop their maintenance dose and demonstrate sustained unresponsiveness on a challenge after weeks to months of avoidance, which was beyond the scope of this study [31].

Serological analysis did show an increase in peanut-specific IgG4 similar to the monotherapy group. Peanut-specific IgE were stable after one year but this was not unexpected, as previous reports have shown that food specific IgE may start decreasing below baseline levels only after the first year of therapy [8,13,16,32].

One limitation to this study was the absence of randomization. This said, this is not a requirement for phase 1 studies. The single and multiple allergic participant were part of the same protocol. We cannot rule out that the molecular sensitization profile could be different in multi-allergic participants that exhibit cross-reactivity with other nuts as it was not tested fully. Regardless, these proof of concept results show that OIT to multiple foods might be as safe as peanut OIT in single-allergic participants.

In conclusion, using a modified OIT protocol we have shown that simultaneous desensitization to multiple foods is feasible and worthy of further study. The reaction profile compared to that of peanut single-allergic participants undergoing monotherapy and participants showed comparable changes on serological examination. At this time, OIT should be considered an experimental treatment and should be conducted by trained research personnel in a hospital setting. Randomized, placebo-controlled phase 2 multicenter trials are needed to continue to determine safety and efficacy parameters of multi OIT in multi-allergic participants.

## Abbreviations

CMC: Chemistry and manufacturing control; DBPCFC: Double-blind placebo-controlled food challenge; IgE: Immunoglobulin E; IND: Investigational new drug; OIT: Oral immunotherapy.

## Competing interests

This project was approved by the IRB committee at Stanford University.

The authors have no relevant conflict of interest to disclose.

## Authors’ contributions

KN conceived and designed the study. PB, LW, TD, LB, TT, BK, AM, AB, GO and MW assessed the patient and acquired clinical data. PB, LW, LB and KN analysed and interpreted the data. MW and AB performed work on food flours/powders. PB, LW and KN drafted the manuscript. All authors revised the manuscript and approved the final version. RH performed serological analyses.

## Supplementary Material

Additional file 1 **Supplemental methods and figures**[33,34] Click here for file
