# Erratum to: Safety and feasibility of oral immunotherapy to multiple allergens for food allergy

**DOI:** 10.1186/s13223-016-0133-1

**Published:** 2016-05-24

**Authors:** Philippe Bégin, Lisa C. Winterroth, Tina Dominguez, Shruti P. Wilson, Liane Bacal, Anjuli Mehrotra, Bethany Kausch, Anthony Trela, Elisabeth Hoyte, Gerri O’Riordan, Scott Seki, Alanna Blakemore, Margie Woch, Robert G. Hamilton, Kari C. Nadeau

**Affiliations:** Allergy, Immunology, and Rheumatology Division, Stanford University, CCSR 3215, Stanford, CA 94305 USA; Johns Hopkins University School of Medicine, Dermatology, Allergy and Clinical Immunology Reference Laboratory, Baltimore, MD USA

## Erratum to: Allergy, Asthma & Clinical Immunology 2014, 10:1 DOI 10.1186/1710-1492-10-1

Unfortunately, the original version of this article [1] contained an error. In the supplemental methods section, lines 86–88 are incorrect.

“Doses were dispensed as one soufflé cup per day containing all the foods mixed together. This mix became was considered and treated as a medication in itself with its own lot number, different from the ones of the various foods that it contained.”

Instead, it should read “The food flours were given separately to the participants. Pursuant to the governmental regulations governing the study, we did not mix the food flours.”
