# Supplementary material for: Safety and feasibility of oral immunotherapy to multiple allergens for food allergy
Source: Allergy Asthma Clin Immunol. 2014 Jan 15;10(1):1. doi: 10.1186/1710-1492-10-1 (PMC3913318; doi:10.1186/1710-1492-10-1)

SUPPLEMENTAL METHODS

Participant selection:

This phase 1 study was performed in a single center in a hospital setting with Institutional Review Board (IRB) approval, under Investigational New Drug (IND) approval. Participants were recruited through advertisement at our institution's as well as local allergy clinics. They were screened for eligibility after signing informed consent at our research unit. They were eligible for inclusion if they: (1) were older than 4 years old; (2) had proven sensitivity to peanut documented by both a skin prick test (with neat extracts from Greer Laboratories, Lenoir, NC) greater than 7mm (wheal), PN-IgE greater than 2ku/L (ImmunoCAP) [E1]; and (3) had clinical reactivity proven by positive allergic reaction in a double-blind placebo-controlled oral food challenge (DBPCFC). Exclusion criteria included: (1) eosinophilic oesophagitis; (2) autoimmune or (3) severe cardiac diseases; chronic treatment with (4) beta-adrenergic antagonists or (5) steroids; (6) a history of severe anaphylaxis requiring admission to an ICU; (7) frequent allergic or non-allergic urticaria; and (8) poorly controlled asthma. DBPCFC was performed on separate days for each food and for the placebo. All participants performed spirometry as appropriate per age and had continuous pulse oximetry monitoring and vital signs checked, before and every 15 minutes after being given increasing doses of placebo (oat flour) or allergenic food protein. Doses were increased following the following scale over 4.5 hours up to a cumulative dose of 182 mg or until an objective reaction occurred. Reaction assessment was based on Bock's criteria and required skin or upper airway objective signs grade 2 or above; expiratory wheezing on auscultation or 15% decrease in FEV1; or at least one episode of emesis or diarrhea [E2].

The doses were as follows:

Dose in mg of protein	Dosing interval in minutes
0.1	15
1.6	30
6	45
25	60
50	60
100	120

Self-administered epinephrine training

Participants underwent a standardised training on the use and indication of self-administered epinephrine at screening, which was reviewed on day one and every 3 months afterward and documented in a log. Training was based on identifying anaphylaxis based on a set of criteria adapted from the World Allergy Organisation [E3].

1. At ANY time: skin or ENT symptoms AND cardiovascular or respiratory symptoms

2. After dose: cardiovascular OR respiratory compromise

3. After dose: skin or ENT symptoms AND persistent abdominal pain or repetitive vomiting

During training, subjects went through all three scenarios with examples including one of progression from mild symptoms. In the event of mild symptoms, parents and participants were instructed to refrain from physical activity and to be kept under strict observation for symptoms that would warrant epinephrine. They were provided examples of cases that escalated from mild reactions and for which new onset severe symptoms had been missed. The training also focused on wheezing, which should always be considered a sign of anaphylaxis after a dose and thus treated with epinephrine rather than albuterol. It was stressed that control of asthma was paramount as uncontrolled asthma is a risk for refractory and fatal anaphylaxis and that they should abstain from dosing if wheezing is present at baseline.

Study medication

This study used only food flours/powders permitted by *Food and Drug Administration* (FDA)-approved GMP guidelines in a phase 1 GMP facility for food allergens at Stanford University/Lucile Packard Children's Hospital. A *Chemistry and Manufacturing Control* (CMC) section was written for each food allergen powder/flour to perform needed assessments for stability, identity, relative sterility, and purity of each of the food powder/flour. These food flours/powders include milk powder (Organic Valley, WI), egg powder (Deb El, NJ), peanut flour (Byrd Mill, VA), walnut flour (Carriere Family Farms, CA), cashew flour (Digestive Wellness, NY), almond flour (Just Almonds, NV), pecan flour (Green Valley, AZ), hazelnut flour (Holmquish Hazelnut Orchards, WA), wheat flour (Gold Medal, MN), soy flour (Honeyville Grain, Inc., UT), and sesame seed flour (Dispasa USA, Inc., TX). For each flour/powder, protein chemistry assays for stability and contamination testing were performed. Each dose was weighted out by a nutritionist on a professional-grade balance. Flour/powder protein content was calculated according to nutritional information provided by manufacturers.

Study design

Participants who reacted only to peanut on their inclusion DBPCFC were assigned to the single OIT group while those who reacted to multiple foods were assigned to multiple food therapy (Figure 1). The multi OIT regimen (between two and five food allergens could be used) was customized to what the participant was found to be allergic to by DBPCFC. For example, if a participant had been through 6 DBPCFC's on separate days (a separate DBPCFC for cashew, sesame, soy, milk, wheat, and placebo; and was found to be allergic to cashew, sesame and soy by DBPCFC, then those three food flours were used in that participant's OIT regimen). The OIT protocol for both groups (single OIT and multi OIT) consisted of three phases: (1) the initial escalation day (or modified rush day), (2) home dosing with biweekly visits for dose escalations

and (3) the maintenance phase (Figure 1). The primary goal of the OIT was to achieve a 10-fold increase from initial DBPCFC threshold. The dosing protocol was designed to continue dose increases up to a daily maintenance dose of 4000mg protein of each allergen (up to 20,000 mg cumulative dose for those on 5 allergens).

Initial Escalation: On the initial escalation day, all participants were admitted to the Clinical Translational Food Unit (CTFU) where their doses were administered by trained clinical staff in a hospital setting, and antihistamines, inhaled beta-2 agonists, solu-medrol and epinephrine were all made readily available at the bedside. The initial dosing began at 0.1 mg protein of each of the offending food allergens (up to five) and doses were slowly increased until the participant reached a dose of 6mg protein (i.e. up to 1.2mg protein of each offending food allergen if the participant's regimen included 5 allergens, or 6 mg protein for a single allergen in monotherapy). Food allergens were given over a period of 3 hours. Participants were monitored every 15 minutes for vital signs and physical assessments throughout the dosing process and were observed for an additional 2 hours after receiving the final dose. The participant's starting daily dose was up to a total dose of 6 mg protein (divided evenly into each of the separate offending food allergen.

Dose in mg of protein	Dosing interval in minutes
0.1	30
0.2	30
0.4	30
0.8	30
1.5	30
3.0	30
6.0	120

Home dosing: Upon confirmation that the dose (up to 6 mg protein of total allergens) could be ingested safely without an allergic reaction, participants received their dose for the following two

weeks to take home. Doses were dispensed as one soufflé cup per day containing all the foods mixed together. This mix became was considered and treated as a medication in itself with its own lot number, different from the ones of the various foods that it contained. Participants were told to ingest their dose after a full meal at approximately the same time each day. Each food allergen was given simultaneously in applesauce or pudding (or another medium the participant had shown tolerance to during placebo challenge). They were instructed not to miss their daily dose. Participants were instructed to take oral cetirizine (dosed as per each product insert) 1 hour before home doses. Pre-dosing with loratadine was also recommended (as per each product insert) if patient reported abdominal symptoms. Participants and their families were given instructions on how to monitor for reactions at home and record any symptoms in their dosing diary. Research staff kept in close contact to proactively investigate any significant adverse events, and participants had 24-hour contact information for all study personnel in case of a significant reaction. All participants were provided with injectable epinephrine devices, oral antihistamines and a treatment plan for possible allergic reactions.

Dose Escalation: The participant returned to the CTFU every two weeks for a dose escalation visit with daily home diaries which detailed any symptoms that occurred and treatments given during the daily home dosing. Staff reviewed the dose diaries with the participants and their families at each visit. A physical examination was performed and asthma control was reassessed by spirometry. If home daily protein flour/powder doses had been well tolerated, the dose was increased in the hospital setting according to a standard scale (previously described in (11)).

Dose in mg of protein	Interval in Weeks	% of Increase from previous
12 mg	2	100%
25 mg	2	108%
50 mg	2	100%
75 mg	2	50%
100 mg	2	33%

125 mg	2	25%
156 mg	2	25%
195 mg	2	25%
245 mg	2	25%
306 mg	2	25%
383 mg	2	25%
479 mg	2	25%
599 mg	2	25%
749 mg	2	25%
936 mg	2	25%
1,170mg	2	25%
1,463mg	2	25%
1,829 mg	2	25%
2,286 mg	2	25%
2,858 mg	2	25%
3,573 mg	2	25%
4,466 mg	2	25%
5,583 mg	2	25%
8,374 mg	2	25%
10,467 mg	2	25%
13,084 mg	2	25%
16,355 mg	2	25%
20,000 mg	2	22%

Participants were monitored by trained clinicians in the CTFU for at least one hour following their new dose. If the new dose was tolerated, it became their daily dose for the following two weeks. Otherwise they continued on their previous dose. Thus, OIT did not advance according to a fixed calendar but rather was individualized according to participants' allergy safety outcomes. There was no limit to the number of attempts at a new dose. According to the investigators assessment, half increases (12.5%) were permitted, especially when external factors, such as environmental allergies, affected the ability to perform a full up-dose. When a new regimen was not tolerated, it was decreased to the previously tolerated dose.

E1. Johannsen H, Nolan R, Pascoe EM, Cuthbert P, Noble V, Corderoy T et al. Skin prick testing and peanut-specific IgE can predict peanut challenge outcomes in preschool children with peanut sensitization. Clin Exp Allergy. 2011 Jul;41(7):994-1000.

E2. Bock SA, Sampson HA, Atkins FM, Zieger RS, Lehrer S, Sachs M, et al. Double-blind, placebo-controlled food challenge (DBPCFC) as an office procedure: a manual. J Allergy Clin Immunol. 1988;82:986–97.

E3. Simons FE, Ardusso LR, Bilò MB, Dimov V, Ebisawa M, El-Gamal YM, et al. 2012 Update: World Allergy Organization Guidelines for the assessment and management of anaphylaxis. Curr Opin Allergy clin Immunol. 2012; 12(4):389-99.

SUPPLEMENTAL TABLES AND FIGURES

TABLE S1 – Baseline allergy tests to other foods in multi-allergic group

Test	MEDIAN	RANGE
**Walnut (n=14)**		
SPT in mm	10	4-14.5
Specific IgE in ku/L	11.3	5.7-52.4
DBPCFC step eliciting symptoms (mg protein)	25	0.1-100
**Cashew (n=13)**		
SPT in mm	13	8.5-25.5
Specific IgE in ku/L	16.5	3.2-76.0
DBPCFC step eliciting symptoms (mg protein)	6	0.1-100
**Pecan (n=7)**		
SPT in mm	9	5.5-12.5
Specific IgE in ku/L	8.6	2.36-169.0
DBPCFC step eliciting symptoms (mg protein)	25	1.6-50
**Milk (n=7)**		
SPT in mm	18.5	5-20.5
Specific IgE in ku/L	11.3	3.6-39.1
DBPCFC step eliciting symptoms (mg protein)	50	25-100
**Sesame (n=6)**		
SPT in mm	12.5	10.5-37.5
Specific IgE in ku/L	23.8	7.1-65.6
DBPCFC step eliciting symptoms (mg protein)	50	6-100
**Egg (n=6)**		
SPT in mm	10.4	7.5-17
Specific IgE in ku/L	11.3	2.6-90.6
DBPCFC step eliciting symptoms (mg protein)	37.5	0.1-100
**Almond (n=5)**		
SPT in mm	5.5	3-12.5
Specific IgE in ku/L	2.45	1.1-3.7
DBPCFC step eliciting symptoms (mg protein)	25	6-100
**Hazelnut (n=3)**		
SPT in mm	18.5	14-21
Specific IgE in ku/L	26.1	13.4-39.1
DBPCFC step eliciting symptoms (mg protein)	25	25-100

TABLE S2 – FOOD COMBINATIONS IN MULTI-ALLERGIC GROUP

Number of foods in mix	Number of participants with mix	Food combination in mix
5	1	Peanut, Walnut, Cashew, Hazelnut, Sesame
5	1	Peanut, Walnut, Pecan, Sesame, Egg
5	1	Peanut, Walnut, Pecan, Cashew, Milk
5	1	Peanut, Walnut, Cashew, Sesame, Almond
5	1	Peanut, Walnut, Cashew, Hazelnut, Almond
5	1	Peanut, Walnut, Cashew, Pecan, Almond
4	1	Peanut, Walnut, Pecan, Hazelnut
4	1	Peanut, Walnut, Milk, Egg
4	1	Peanut, Walnut, Pecan, Cashew
4	1	Peanut, Walnut, Cashew, Sesame
4	1	Peanut, Sesame, Milk, Egg
3	3	Peanut, Milk, Egg
3	1	Peanut, Walnut, Pecan
3	1	Peanut, Walnut, Milk
3	1	Peanut, Pecan, Cashew
3	1	Peanut, Walnut, Sesame
3	1	Peanut, Walnut, Cashew
2	4	Peanut, Cashew
2	2	Peanut, Almond

FIGURE S1 – REACTION PROFILE IN MONO-ALLERGIC PARTICIPANTS. Symptom occurrence with (A) initial escalation day, (B) dose escalations and (C) home dosing during OIT to multiple foods.

A

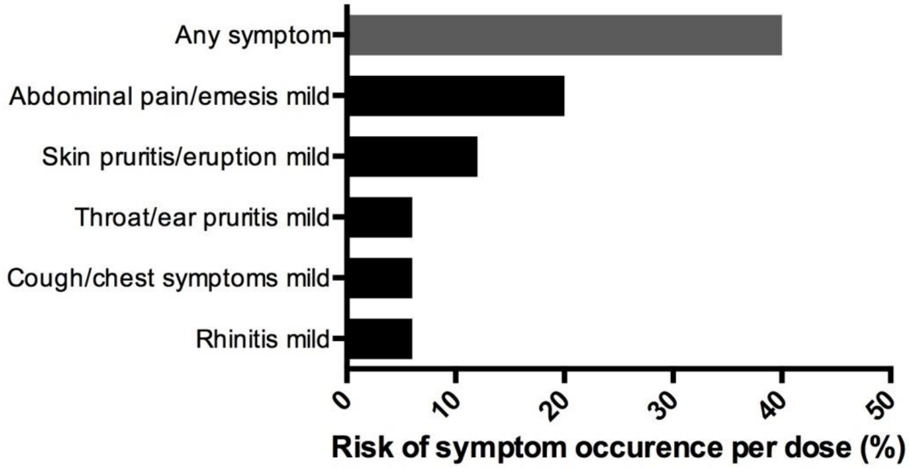

B

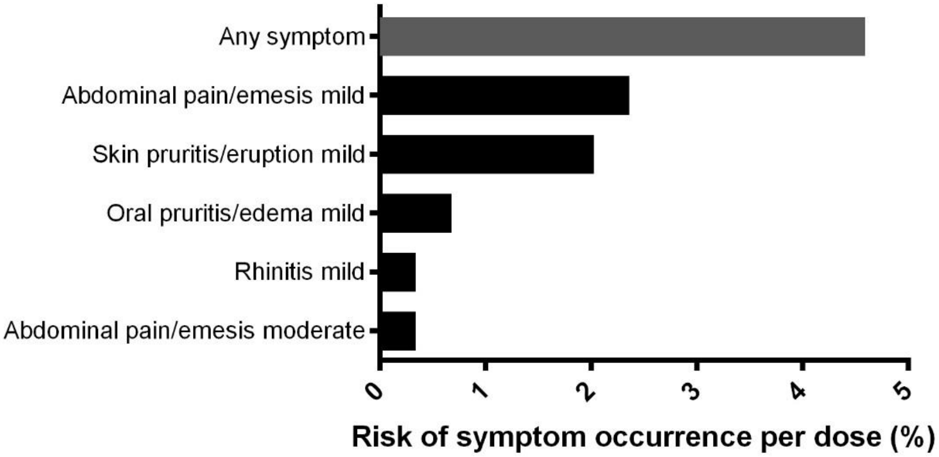

C

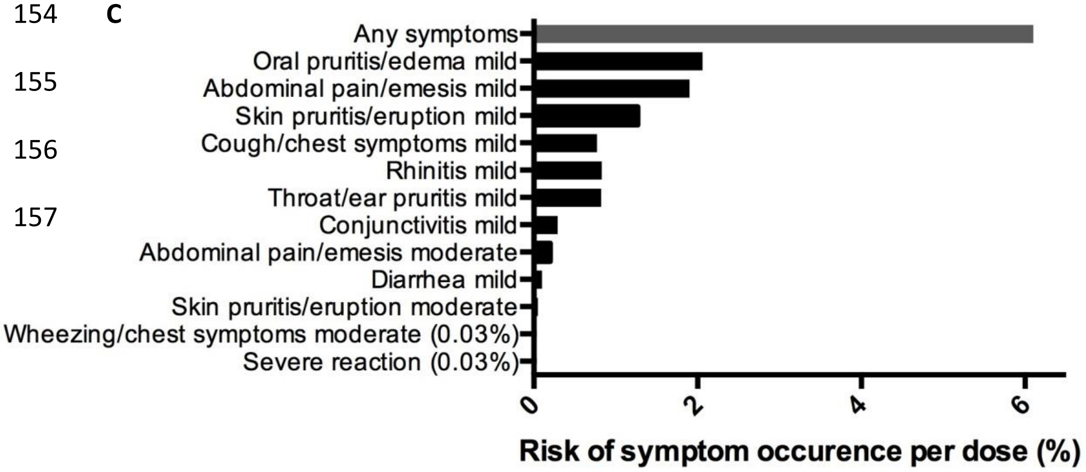

Table S3 – EPINEPHRINE USES

Participant	A	B	C	D
**Group**	Single OIT	Single OIT	Multi OIT	Multi OIT
**Context**	Home dosing	Home dosing	Home dosing	Home dosing
**Dose (mg protein)**	936 mg	1170 mg	10903 mg	599 mg
**Allergens in mix**	Peanut	Peanut	Peanut, Walnut, Cashew, almond, hazelnut	Peanut, Milk, Egg
**Symptoms**	Abdominal pain, urticaria, wheezing	Urticaria, wheezing	Abdominal pain, urticaria, wheezing	Abdominal pain, angioedema around the eyes, wheezing
**Time elapsed between:**				
**Dosing and symptoms**	20 minutes	40 minutes	35 minutes	25 minutes
**Epinephrine and resolution**	6 minutes	5 minutes	6 minutes	3 minutes

FIGURE S2 – SEROLOGICAL ANALYSES FOR OTHER FOODS IN MULTI-SENSITIZED GROUP

Comparison of peanut-specific IgE (A) and IgG4 (B) at baseline and after one year of OIT. *p=0.016

A

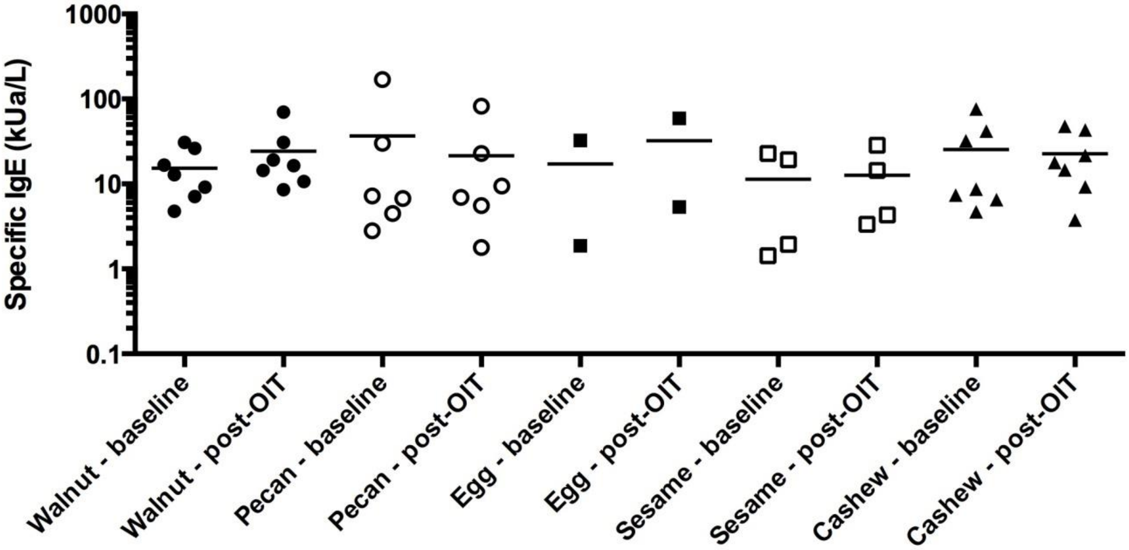

B

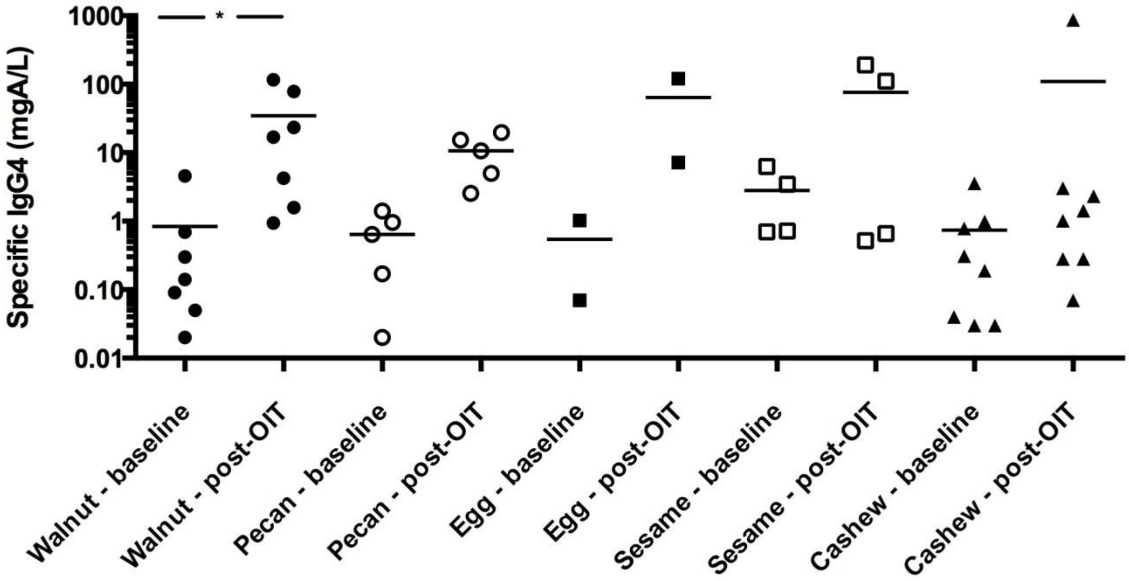

Supplement: Additional file 1 — Supplemental methods and figures[33,34] [file 1710-1492-10-1-S1.pdf]
